# Development of a deformable lung phantom for the evaluation of deformable registration

**DOI:** 10.1120/jacmp.v11i1.3081

**Published:** 2010-01-28

**Authors:** Jina Chang, Tae‐Suk Suh, Dong‐Soo Lee

**Affiliations:** ^1^ Dept. of Biomedical Engineering College of Medicine Catholic University Seoul Korea; ^2^ Dept. of Nuclear Medicine Seoul National University Hospital Seoul Korea

**Keywords:** deformable lung phantom, deformable registration, evaluation

## Abstract

A deformable lung phantom was developed to simulate patient breathing motion and to evaluate a deformable image registration algorithm. The phantom consisted of an acryl cylinder filled with water and a latex balloon located in the inner space of the cylinder. A silicon membrane was attached to the inferior end of the phantom. The silicon membrane was designed to simulate a real lung diaphragm and to reduce motor workload. This specific design was able to reduce the use of metal, which may prevent infrared sensing of the real position management (RPM) gating system for four‐dimensional (4D) CT image acquisition. Verification of intensity based three‐dimensional (3D) demons deformable registration was based on the peak exhale and peak inhale breathing phases. The registration differences ranged from 0.60 mm to 1.11 mm and accuracy was determined according to inner target deformation. The phantom was able to simulate features and deformation of a real human lung and has the potential for wide application for 4D radiation treatment planning.

PACS number: 87.57.Gg

## I. INTRODUCTION

Adaptive radiation therapy (ART) is a replanning procedure that conforms to organ changes that occur due to breathing, weight loss, or tumor shrinkage during the course of treatment. Various techniques have contributed to the success of ART including deformable registration, automatic segmentation, and dose accumulation.^(^ ^1^ ^–^ ^2^ ^)^ However, verification of these techniques is a difficult task for the quantification of anatomic variation and requires the use of a competent deformable phantom.

Several investigators have sought to develop a reproducible, deformable, tissue‐equivalent phantom to perform accurate assessment of respiratory‐correlated movements. We have previously fabricated a phantom to simulate organ motion using simple moving targets^(^ ^3^ ^–^ ^4^ ^)^ and an inflatable balloon.^(5)^ However, for an accurate clinical application, a phantom is needed to represent real organ structures and densities.

Nioutsikou et al.^(6)^ have developed a sophisticated deformable phantom for lung tumor dosimetry. An accordion‐type flexible bottle that simulated a lung was described where dosimetric film was inserted. Kashani et al.^(7)^ have designed a tissue‐equivalent deformable lung phantom using a commercial diagnostic thoracic phantom as the main shell and high density foam with tumor‐simulating insets as the inner lung material. The investigators used this phantom to verify the use of the thin‐plate spline deformable registration technique.^(8)^ However, this phantom was insufficient to simulate a real lung and tumor deformation and, consequently, the phantom had a limited ability to provide accurate deformable registration. Serban et al.^(9)^ have developed an anthropomorphic lung phantom that consisted of a latex balloon with dampened sponges and a piston that simulated the thoracic cavity and diaphragm. The balloon and water in the space around the balloon were compressed and were decompressed by a piston that was fastened to a programmable motor. This tissue‐equivalent phantom simulated real lung deformation according to breathing patterns. However, a certain amount of metal was used to support the phantom and to exert a large force on the piston without water leakage. The metal may cause a metal artifact on CT scans and may prevent infrared sensing of the real position management (RPM) gating system for four‐dimensional (4D) CT image acquisition.

We have also developed an anthropomorphic deformable lung phantom by the use of a latex balloon containing various elastic materials and a silicon membrane. The elastic materials were used as landmarks for deformable registration. The silicon membrane that simulates the diaphragm serves as a medium to improve motor efficiency and minimize metal use.

The demons deformable registration algorithm has been used to register lung CT scans. Guerrero et al.^(10)^ have applied a three‐dimensional (3D) optical flow method to validate intrathoracic tumor motion estimation. Wang et al.^(11)^ have used an accelerated demons algorithm and have evaluated the algorithm for prostate, head‐and‐neck, and lung cases. Wu et al.^(12)^ reported an average alignment error reduction of 2.07 mm using the demons algorithm with boundary‐matching penalty on 4D CT data sets. In this study, validation of demons deformable registration was performed based on distance and vector differences between 3D reference and deformed image sets. We have used a phantom for verification of the demons deformable registration algorithm.

## II. MATERIALS AND METHODS

### A. Experimental setup

**Figure 1:**
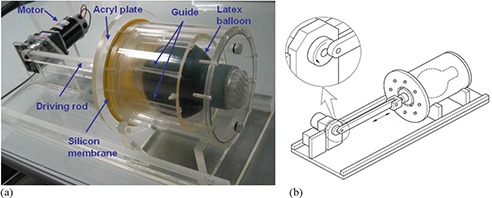
Deformable lung phantom: (a) photograph; (b) diagram.

The anthropomorphic lung phantom shown in Fig. 1(a) was designed to simulate real lung deformation. The phantom consisted of an acryl cylinder filled with water. A latex balloon was located inside the inner space of the cylinder. The diameter and length of the acryl cylinder were 22 cm and 24 cm, respectively. The latex balloon simulated the thoracic cavity, and the balloon was filled with sponges and targets where the targets were embedded in the sponges. Each target was made of a deformable water balloon and silicon and a rigid glass ball.

**Figure 2:**
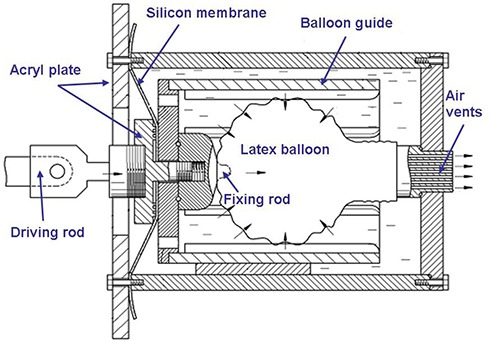
Diagram of the phantom specifications.

A silicon membrane was attached at the inferior end of the phantom. The silicon membrane was designed to simulate a real lung diaphragm and to reduce the motor workload. This specific design was able to reduce the motor size of the phantom. An acryl plate was attached to the driving rod to distribute power uniformly to the diaphragm. Since the diving rod was attached the wheel of the motor as shown in Fig. 1(b), the strain gauge of the real‐time position management (RPM) system can be used to obtain a series of CT images. The motor was programmed for regular breathing patterns with various breathing periods. When power was applied to the diaphragm, the water in the phantom pushed a latex balloon and the air in the balloon flowed out of air vents (Fig. 2). A balloon guide and fixing rod were designed to guide the balloon to a designated location. The device was designed to improve phantom reproducibility.

CT image sets were acquired using the RPM respiratory gating system from Varian Medical Systems (Varian, Palo Alto, CA USA) and a Philips CT scanner (Philips, Milpitas, CA USA). The image resolution was $0.7\times 0.7\times 2.5\;{\text{mm}}^{3}$ and a total of ten 3D CT image sets were obtained according to ten different phases.

### B. Demons deformable registration

For this study we have used the intensity‐based demons deformable registration algorithm.^(13)^ The demons algorithm is suitable for deformable registration for images acquired with the same modality, as the algorithm assumes that the voxels are homologous by representation of the same points that have equal intensity on both images. The 3D variant demons algorithm implemented in the Insight Toolkit (ITK)^(14)^ was used to calculate a deformation grid. More specifically, before processing an image fusion, the CT image sets were converted to the 3D mhd file format (i.e. a meta image header file) with the same pixel spacing and thickness. The demons algorithm is based on gradient calculations from both static and moving images to determine the demons force. The displacement between images *D* is calculated from

(1) $$D=-\frac{2(m-f)(\nabla f+\nabla g)}{|\nabla f+\nabla g{|}^{2}+{\left(m-f\right)}^{2}}$$

where *m* and *f* are moving and fixed images, respectively, and ∇f and ∇g are gradients of the fixed and moving images, respectively. Linear interpolation for the 3D resampling procedure was used to compute values at non‐integer positions for the original CT image.

Exhale data in 3D CT image sets was the moving model and deformable registration was performed to align the moving model to the static reference model (the inhale data set). Registration accuracy was measured with the center of gravity (COG) parameter. The volume of targets was defined separately on moving image, and reference image sets and COGs were calculated. The distances of the COGs were compared. We are aware that the edge of a tumor is also an important factor to account for uncertainties associated with patient breathing and to determine planning target volume expansion. However, we only measured the COG for quantitative registration evaluation. The COGs were calculated based on the use of the CorePLAN radiation planning system (Seoul C & J, Seoul, Korea).

## III. RESULTS

**Figure 3:**
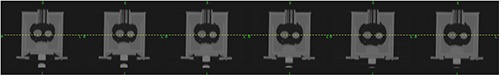
Three‐dimensional CT image sets between peak exhale and peak inhale.

**Figure 4:**
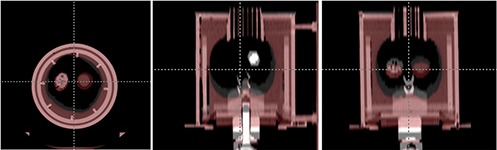
The results of rigid body registration between peak exhale and peak inhale.

**Figure 5:**
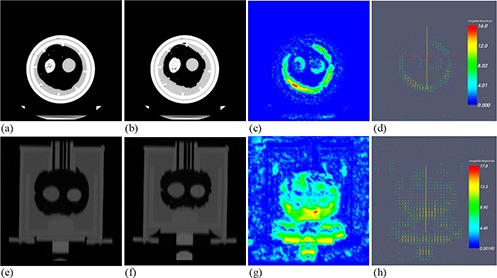
Results for deformable registration for: (a) peak inhale, (b) peak exhale, (c) the deformable magnitude, and (d) vector (seen on an axial view); and for: (e) peak inhale, (f) peak exhale, (g) the deformable magnitude, and (h) vector (seen on a coronal view).

Figure 3 shows the 3D CT image sets acquired for the corresponding respiratory cycles. This figure depicts amplitude differences for different phases between peak inhale and peak exhale. The peak inhale to peak exhale distance of the silicon membrane was 2 cm, and the maximum volume and minimum volume were 1505.6 cc and 1111.1 cc, respectively. Figure 4 shows the results of rigid body registration. It demonstrates volume change and target movement between peak inhale and peak exhale on axial, sagittal, and coronal views. Figure 5 shows the results of deformable registration. Figures 5(a) and (b) show peak inhale and exhale images for axial views, while Figures 5(c) and (d) show the deformable magnitude and vector after execution of the demons deformable registration algorithm. The space around the silicon balloon is filled with water, which replicates a chest mass in the phantom. The presence of water affected the strong contraction of the lower area of the balloon when power was applied, due to gravity. This effect is not relevant for a real patient as the chest mass of the patient is composed of muscles. Figures 5(e) and (f) show peak inhale and exhale images for coronal views. Figures 5(g) and (h) show the deformable magnitude and vector after execution of the demons deformable registration algorithm. The displacement of the target as seen on a coronal view is relatively small as compared to the displacement of the silicon membrane due to the flexibility of the sponges.

For the quantitative evaluation of deformable registration, we calculated the COGs of the phantom. Table 1 represents the residual differences between deformed image and reference image sets. The static target point was normalized as reference point 0 and was responsible for demonstrating 3D vector differences. As shown in Table 1, the vector differences ranged from 0.60 mm to 1.11 mm from the glass target to the water balloon target, and the registration accuracy was determined according to target deformation. The water balloon, which had a larger deformation, showed a larger discrepancy; the rigid glass ball showed relativity good agreement within 1 mm of the results for image registration accuracy.

**Table 1:** Residual differences between deformed image and reference image sets.

*(mm)*	*Target 1 (water balloon)*	*Target 2 (silicon)*	*Targt 3 (glass)*
x‐translation (lateral)	0.14±0.05	0.01±0.06	0.01±0.03
y‐translation (longitudinal)	−0.08±0.09	0.13±0.11	0.08±0.06
z‐translation (vertical)	1.09±0.60	0.98±0.44	−0.60±0.21
3D vector difference	1.11±0.60	1.04±0.33	0.60±0.20

## IV. CONCLUSIONS

We have developed an anthropomorphic deformable lung phantom including specific structures for phantom efficiency and reproducibility, and have verified demons deformable registration. The accuracy of the registration was influenced by deformation of the inner target material. A deformable phantom should contain various deformable targets for the accurate evaluation of deformable registration. Validation of deformable registration was performed based on distance and vector differences between 3D reference and deformed image sets. An advantage of this evaluation method is convenient implementation for most radiation treatment planning systems.

This phantom could simulate the features and deformation of a real human lung, and has a wide potential for lung ART including 4D radiation treatment planning. In addition to the verification of deformable registration, the phantom can be used to determine whether there is a dosimetric advantage to compensate doses by calculation of the cumulative dose distribution during the course of radiation treatment. Moreover, this phantom can be used to evaluate a 4D CT reconstruction algorithm and dosimetric uncertainties due to organ motion and deformation. Further studies will be needed to evaluate long‐term reproducibility and the reliable application of deformable phantoms.

## ACKNOWLEDGEMENTS

This research was sponsored by the Seoul R&BD Program (10550C0211604) and the Nuclear R&D Program of the Ministry of Education, Science and Technology (20090062514).
